# Veratrum Veride, a Sure Antidote to Opium Poisoning

**Published:** 1880-08

**Authors:** 


VERATRUM VERIDE, A SURE ANTIDOTE TO OPIUM POISONING.—We made some statements, editorially, in the last number of the *Independent Practitioner*, in regard to a prescription of equal quantities of veratrum veride and laudanum in *pneumonia*, by a certain "professor;" and we desire now to place upon record, not only the ascertained fact that veratrum veride is one of the best, if not the very best antidotes in recent opium poisoning, but has been found useful in *destroying* the "opium habit." Dr. J. L. Holdeman, of Zanesville, O., after stating the *general incompatibility* of opium, and veratrum, in recent cases says, viz: "The two articles, opium and veratrum, have evidently antipodal effects, as much so perhaps, as *any other two* in the materia medica;" and then goes on to establish, in a late number of the *Cincinnati Lancet and Clinic*, the value of veratrum in "chronic opium inebriety." Time will not allow of our saying more in this connection now, but we feel sure that the subject will receive the attention it demands at the hands of the profession generally.

